# Vitamin A and D levels in patients with filarial lymphedema and healthy volunteers in Puducherry, India

**DOI:** 10.3389/fnut.2025.1610014

**Published:** 2026-01-08

**Authors:** Shakila Venkatesan, Anbusivam Sadhasivam, Vijayalakshmi Gnanasekaran, Madhumidha Periyathambi, Hisham Moosan, Vijesh Sreedhar Kuttiatt

**Affiliations:** 1 Unit of Clinical and Molecular Medicine, ICMR-Vector Control Research Centre (VCRC), Puducherry, India; 2 Model Rural Health Research Unit (MRHRU), Community Health Centre Campus, Karikalampakkam, Puducherry, India; 3 ICMR-Vector Control Research Centre, Field Station, Kottayam, Kerala, India

**Keywords:** filarial lymphedema, morbidity management and disability prevention, global program for elimination of lymphatic filariasis, micronutrients, vitamin A, vitamin D

## Abstract

Lymphatic filariasis (LF) is a parasitic disease caused by filarial nematode worms, transmitted by mosquitoes. Infection damages the lymphatic system which leads to limb lymphedema and hydrocele. LF continues to remain one of the major neglected tropical diseases. Patients with filarial lymphedema are prone to nutritional deficiencies due to poverty, poor nutrition, old age, and frequent adenolymphangitis. However, studies on micronutrient deficiencies in affected patients remain scarce. In this study, we aimed to assess the levels of vitamin A and D in patients visiting a specialized clinic for filarial lymphedema management. We recruited 40 patients and analyzed their vitamin A and D levels. In parallel, we also evaluated a set of 30 apparently healthy volunteers. Sociodemographic and clinical data were collected through a semi-structured interview using a proforma. Dietary data were recorded using a 24-h dietary recall method. Among 40 patients with filarial lymphedema, three had vitamin A deficiency, and so did six among the 30 healthy volunteers. Twenty patients and equal number of healthy volunteers were deficient in vitamin D. The study highlights vitamin D deficiency among patients with filarial lymphedema and healthy individuals. It is desirable to have nutritional counseling and multivitamin supplementation in the Morbidity Management and Disability Prevention (MMDP) component of the LF elimination program. Creating awareness about vitamin D deficiency among health care workers and public is also essential.

## Introduction

Lymphatic filariasis (LF) is a neglected tropical skin disease (skin NTD) caused by the filarial worms, *Wuchereria bancrofti, Brugia malayi and Brugia timori*. 90% of cases worldwide are caused by *W. bancrofti* (1). Infection may lead to lymphedema or elephantiasis of the limbs, as well as hydrocele. LF is a disease of the poor and is the most common cause of irreversible disability globally. It affects 120 million people in 72 countries, and India accounts for 40% of the global prevalence of infection (2). In 2021, India reported 525,440 cases of lymphedema and 144,645 cases of hydrocele, primarily from the states of Bihar, Jharkhand, Odisha, Telangana, and Uttar Pradesh. India targets the elimination of LF by 2027, 3 years ahead of the global goal of 2030 (3).

World Health Organization (WHO) initiated the Global Program to Eliminate Lymphatic Filariasis (GPELF) in 2000, which focuses on interrupting transmission through Mass Drug Administration (MDA) and Morbidity Management and Disability Prevention (MMDP) program caters to the needs of patients affected with lymphedema (4). The mainstay of management of lymphedema is supportive, which includes ensuring limb hygiene, regular exercises, massage and compression to improve lymphatic drainage. Affected persons suffer from recurrent skin and soft tissue infections termed adenolymphangitis, leading to further disease progression. Skin infections are amenable to treatment by antibiotics. MMDP practices are helpful in the prevention of ADL episodes and disease progression to a great extent.

Nutritional deficiencies are common among people from the low socioeconomic class in tropical regions. Malnutrition causes immunodeficiency and increases the susceptibility to infection. Infection, in turn, leads to malnutrition, and thus, it is a vicious cycle with a bidirectional relationship (5). Micronutrients play a significant role in health and diseases. Micronutrient deficiency may occur in patients with filarial lymphedema due to poor nutritional intake, or the disease condition may induce deficiency. A study from Ghana reported the presence of widespread malnutrition among patients with filarial lymphedema (6). Because of the neglected status of filariasis, studies on micronutrient deficiencies in affected patients remain scarce. We sought to evaluate vitamin A and D levels—two micronutrients essential for healthy skin, in patients with filarial lymphedema attending our clinic.

Vitamin A, a fat-soluble vitamin, is primarily sourced from natural foods such as liver, egg yolks, fish, dairy, vegetables, and fruits. Retinoic acid, derived from vitamin A, is essential for the homeostasis of epithelial tissues and mucosa. It regulates gene expression in skin tissues through its interaction with retinoic acid receptors. Hence, deficiency of vitamin A leads to unhealthy skin and increased susceptibility to infections and inflammation (7). All-trans retinoic acid (atRA), the active form of vitamin A, plays a key role in development and immune regulation by binding to the nuclear receptor RARα. Recent studies show that atRA can reduce Th2 cell activity. It lowers the expression of Th2-related transcription factors like Spi1 and cMaf, without affecting GATA3. atRA also decreases the production of IL-4 and IL-13, while increasing IL-5, likely through upregulation of GFI-1. These findings suggest that atRA helps suppress Th2 responses by regulating key genes and transcription factors, highlighting its potential as a therapeutic agent in type 2 immune-related conditions (8).

Vitamin D is another fat-soluble vitamin naturally obtained from foods like fatty fish, cod liver oil, egg yolks and fortified milk (9). Vitamin D is essential for various physiological functions in the body. It exists mainly as ergocalciferol (vitamin D₂) in plant sources and cholecalciferol (vitamin D₃) in animal sources. The main source of vitamin D for humans is the skin where it’s production is triggered when exposed to sunlight. This process starts with the transformation of 7-dehydrocholesterol (7-DHC) into pre-vitamin D3 due to UVB radiation, and then it undergoes thermal isomerization to become cholecalciferol (vitamin D3). The function of vitamin D for a long time, was considered the maintenance of skeletal architecture through calcium homeostasis, but in the last few decades, extra skeletal effects of vitamin D have become apparent, and its role in the regulation of cell proliferation is better understood (10).

Vitamin D, in its active form known as calcitriol, is crucial for the regulation of both the innate and adaptive immune systems. It boosts the innate immune response by stimulating the production of antimicrobial peptides like cathelicidin, thereby enhancing the body’s defense against pathogens. Furthermore, vitamin D contributes to immune equilibrium by decreasing pro-inflammatory T helper cells (Th1, Th17) while increasing regulatory T cells, which help to avert autoimmune reactions. These immunomodulatory effects underscore the potential of vitamin D in the prevention and management of autoimmune and inflammatory diseases (11).

Vitamin A and D deficiencies may contribute to poor skin health as well as immune dysfunction. This may further exacerbate the susceptibility to frequent ADL attacks. Identifying and treating correctable co-morbidity conditions is an important aspect of management of any chronic disease. In this background, we conducted a study to find whether patients with filarial lymphedema attending our Filariasis Management Clinic suffer from deficiencies of these two vitamins essential for healthy skin. In parallel, we also evaluated a group of 30 healthy volunteers.

## Methods

### Study design and setting

This study was conducted at the Filariasis Management Clinic of the ICMR-Vector Control Research Centre, Puducherry. Patients with filarial lymphedema above 18 years of age and belonging to any gender were enrolled. Participants who refused to give their consent and people having acute medical problems or who underwent any recent major surgeries were not considered. In parallel, we also evaluated 30 healthy volunteers who were staff of our institute. All of them were free of lymphedema.

### Data collection

A semi-structured interview was conducted using a proforma and the personal, demographic, dietary and clinical details were recorded. Anthropometric measurements - height, weight and body mass index as well as clinical findings were noted down. Dietary data were recorded using a 24-h dietary recall method. The daily intake was calculated using an online software NutriSurvey (12). Data were entered in electronic spreadsheets.

### Biochemical assays

Five milliliters of venous blood sample was collected from all participants. Serum vitamin A level was measured using a high-performance liquid chromatography (Shimadzu, Japan). Cut off levels were defined for deficiency of vitamin A and D based on WHO criteria. A plasma or serum retinol concentration <0.70 μmol/L indicates subclinical vitamin A deficiency in children and adults, and a concentration of <0.35 μmol/L indicates severe deficiency (13). Serum vitamin D level [25(OH)D] was measured using chemiluminescence assay (Beckman Access 2 CLIA analyzer, California, United States). Serum 25(OH)D concentration less than 30 nmol/L is defined as deficiency and levels between 30 and 50 nmol/L (12–20 ng/mL), as inadequacy. Levels of 50 nmol/L (20 ng/mL) or above are considered as sufficient (14). Assays for vitamin A and D were performed in a National Accreditation Board for Testing and Calibration Laboratories (NABL) accredited lab.

### Statistical analysis

All data were tabulated and analyzed using IBM SPSS 22 software. Categorical variables were described as frequencies and percentages and compared using Fisher’ exact test. Quantitative variables with normal distribution were described by the mean and standard deviation and compared between the groups by Student’s t test for independent samples and *p* value of <0.05 was kept as significant. Normality of the data was tested using Kolmogorov Smirnov test.

## Results

A total of 40 patients with filarial lymphedema and 30 apparently healthy volunteers were enrolled in the study. Mean age of the patients was 57.8 (±10.5) years. There were 21(52.5%) males and 19(47.5%) females. The majority of the participants belonged to the lower socio-economic status with below secondary school education. A total of 30 healthy volunteers were recruited. There were 16 males (53.3%) and 14 females (46.7%). Most of the participants belonged to 50–60 years of age group (56.7%). Mean age was 51.2 ± 5.5 years. Table 1 summarizes the socio-demographic characteristics, anthropometric parameters, lifestyle, comorbidities of patients and controls. The dietary details are summarised in Table 2.

**Table 1:** Characteristics of patients and controls.

Characteristics	Patient	Control	*P*-value
	(*n* = 40)	(*n* = 30)	
Gender			
Male	21 (52.5)	16 (53.3)	
Female	19 (47.5)	14 (46.7)	0.945
Age (in years)			
<40	3 (7.5)	1 (3.3)	
40–50	7 (17.5)	12 (40)	0.001
50–60	14 (35)	17 (56.7)	
>60	16 (40)	0	
Mean age ± SD	57.8 ± 10.5	51.2 ± 5.5	<0.001
Minimum-maximum	35–76	40–59	
Education			<0.001
Below secondary school	35 (87.5)	13 (43.3)	
Above secondary school	5 (12.5)	17 (56.7)	
House type			
Pucca	28 (70)	24 (80)	
Semi pucca	10 (25)	5 (16.7)	0.638
Kaccha	2 (5)	1 (3.3)	
Socio economic status*			
Upper class	0	2 (6.7)	
Upper middle class	6 (15)	14 (46.6)	0.001
Lower middle class	10 (25)	2 (6.7)	
Upper lower class	15 (37.5)	12 (40)	
Lower class	9 (22.5)	0	
Body mass index			
Underweight (<18.50)	1 (2.5)	2 (6.7)	
Normal (18.50–22.99)	6 (15)	5 (16.7)	0.633
Overweight (23–24.99)	4 (10)	5 (16.7)	
Obese (≥25.00)	29 (72.5)	18 (60)	
Smoking habits**	6 (28.6)	2 (12.5)	0.239
Alcohol consumption**	7 (33.3)	7 (43.8)	0.517
Tobacco chewing	5 (12.5)	1 (3.3)	0.175
Menstrual history^#^			
Menstruating	2 (10.5)	4 (28.6)	0.184
Menopause	17 (89.5)	10 (71.4)	
Hypertension	11 (27.5)	3 (10)	0.063
Diabetes mellitus	7 (17.5)	4 (13.3)	0.635
Hypothyroid	2 (5)	1 (3.3)	0.733
Lymphedema grade			
Grade 2	14 (35)	–	–
Grade 3	11 (27.5)		
Grade 4	15 (37.5)		

SD, Standard deviation; *Socio-economic status classified as per modified Kuppuswamy scale (27). **Data presented in the table related to male participants. ^#^indicates female participants.

**Table 2:** Dietary details of patients and controls.

Dietary pattern	Patient	Control	*P*-value
	Frequency n (%)		
Vegetarian	2 (5)	4 (13.3)	0.218
Non-vegetarian	38 (95)	26 (86.7)	
Vitamin supplements	32(80)	2 (6.7)	<0.001

### Vitamin A and D levels in patients and controls

A summary of the vitamin A and D levels observed in patients and healthy volunteers is provided in Table 3. The mean levels of vitamin A was found to be 0.39 ± 0.16 (mg/L). Among a total of 40 patients, 7.5% (3) were found to be deficient in vitamin A. Mean level of vitamin A in healthy volunteers were 0.30 ± 0.13(mg/L). 20% (*n* = 6) were found to be deficient of vitamin A.

**Table 3:** Vitamin A levels and Vitamin D levels in patients and controls

**Vitamin**	**Parameter**	**Patient group [n=40]**	**Control group [n=30]**	*P*-value
**Vitamin A (mg/L)**	Mean ± SD	0.39 ± 0.16	0.30 ± 0.13	0.021
	Deficient, n (%)	3 (7.5)	6 (20)	0.122
	Not deficient, n (%)	37 (92.5)	24 (80)	
**Vitamin D (nmol/L)**	Mean ± SD	55.84±21.05	45.92±12.97	0.018
	Low, n (%)	20 (50)	20 (66.7)	0.071
	Not deficient, n (%)	9 (22.5)	1 (3.3)	

Mean levels of 25 OH vitamin D in patients was 55.84 ± 21.05(nmol/L). 50% (20) of the patients were found to be deficient while 22.5% (*n* = 9) were insufficient in vitamin D. Mean levels of 25 OH Vitamin D in healthy volunteers were 45.92 ± 12.97(nmol/L). 66.7% (*n* = 20) were found to be deficient in vitamin D. There was no significant difference observed between two groups (Table 4).

**Table 4:** 24-h dietary recall data for patients and controls.

Components	Patient	Control	*P*-value
	Mean ± SD	Mean ± SD	
Calories			
Male (2,425 kcal/d)	2277.21 ± 415.54	2393.05 ± 460.15	0.428
Female (1875 kcal/d)	2040.92 ± 522.70	1981.96 ± 315.61	0.711
Carbohydrate (500 g/d)	431.74 ± 103.84	438.14 ± 85.64	0.784
Protein			
Male (60 g/d)	61.36 ± 15.28	70.11 ± 28.60	0.239
Female (50 g/d)	59.78 ± 15.65	58.05 ± 13.68	0.743
Fat (20 g/d)	27.80 ± 15.69	26.12 ± 13.69	0.642
Vitamin A (2,400 μg/d)	1056.44 ± 1331.21	1362.69 ± 1665.57	0.396

### Results of logistic regression analysis

Vitamin A deficiency was more common in females (66.7%), individuals aged 50–60 years (66.7%), overweight/obese (77.8%), and those with education below 10th standard (55.6%). It was also higher among the upper lower socio-economic class (66.7%), non-vegetarians, and non-users of vitamin supplements, though none of these associations were statistically significant (*p* > 0.05).

Vitamin D deficiency was also more prevalent in females (57.5%), the overweight/obese (80%), less educated individuals (62.5%), and the 50–60 age group (37.5%). A significant association was observed with smoking (*p* = 0.049), where non-smokers had higher rates of normal vitamin D status.

Other factors such as alcohol use, tobacco chewing, diet, comorbidities, and lymphedema grade showed no statistically significant associations with either deficiency.

## Discussion

The current study examined vitamin A and D levels in patients with filarial lymphedema attending a special clinic for filariasis management in Puducherry, southern India. Most of the patients had adequate vitamin A levels, whereas most had insufficient vitamin D levels. A parallel evaluation carried out in healthy volunteers showed that most had adequate vitamin A levels, whereas insufficient levels of vitamin D were noted in the majority.

Vitamin A deficiency in children is a known problem, and a national program exists in India to address this. However, data among adults are scant. We found vitamin A deficiency in 7.5% of the patients and 20% of the healthy volunteers. At the same time, 50% of the patients and 66.7% of the healthy volunteers were found to be deficient of vitamin D.

In contrast to our expectation, vitamin D levels were found to be lower among healthy controls enrolled in the study than in patients. This could be due to multiple reasons. A significant proportion of patients were receiving vitamin supplements (80%), while only 6.7% of controls were on supplements. This may be one of the reasons for the observed difference. Also, the healthy volunteers were our staff, and most were working in the office, and the sun exposure levels might be low. Dietary factors also might have contributed; patients were taking vitamin D-rich foods compared to healthy volunteers. Other factors like body composition and inflammatory status might influence vitamin D metabolism and bioavailability. These possibilities highlight the complexity of vitamin D regulation and underscore the need for further investigation through longitudinal studies incorporating detailed lifestyle, dietary, and biochemical assessments to clarify the underlying mechanisms.

To our knowledge, there are no previous published studies that analyzed vitamin A levels in patients suffering from filarial lymphedema. However, there are a few studies that examined vitamin A levels in patients with active LF infection. Friis et al., investigated the impact of *W. bancrofti* microfilarial intensity on micronutrient status, specifically a-tocopherol (vitamin E), b-carotene and retinol. It was found that higher microfilarial intensity was a strong negative predictor of a-tocopherol levels and showed a marginal negative association with b-carotene and retinol. This may reflect increased oxidative stress resulting from the host–parasite interaction, which could lead to depletion of antioxidant micronutrients such as a-tocopherol b-carotene and retinol, potentially contributing to the pathogenesis (15).

In a recent review, Gombart et al. highlights vitamin A as a key micronutrient for maintaining body’s physical barriers, like the skin and the lining of the gut, which protect from harmful microbes. It supports immune cells such as macrophages and natural killer (NK) cells, helping them to fight infections more effectively. Vitamin A has a role in reducing excessive inflammation and it support the production of protective antibodies, especially in the gut. Deficiency of vitamin A increases the risk of infections like diarrhea, measles, and respiratory illnesses (16). In a study by Amewu et al. among individuals clinically diagnosed with LF in the Ahanta West District, southeastern Ghana, vitamin A was found to be deficient in all participants (17). In another study by Nielsen et al. examined micronutrient status indicators in individuals infected with either HIV, *W. bancrofti*, or both, before and after DEC treatment (18). They found a negative correlation between HIV infection and antioxidant vitamins, such as b-carotene and a-tocopherol and that likely stems from oxidative stress induced by the infection. On the other hand, *W. bancrofti* infection does not seem to create considerable oxidative stress. The observed decline in antioxidant vitamins after treatment may be attributed to oxidative stress from HIV progression and inflammation caused by the death of adult worms and microfilariae in *W. bancrofti* infection, rather than an effect of DEC treatment itself (18).

In a study by Dwivedi et al., vitamin D was administered to LF patients, and it was found that levels of cytokines, including TNF alpha and IL-6, were significantly reduced (19). There was a reduction in counts of total leukocytes and polymorphonucleocytes from baseline to follow-up (post vitamin D supplementation), indicating a possible role of vitamin D supplementation over containment of systemic infection. There are studies in India which examined vitamin D levels in healthy populations as well. A systematic review by Sandhiya and colleagues assessed vitamin D levels in apparently healthy populations across different regions of India. The average serum 25-OH vitamin D among Indians was 14.16 ng/mL (95% CI, 13.27, 15.05). The study also revealed regional differences within India. Specifically, South Indians exhibited a higher average serum 25-OH vitamin D, level 17.45 ng/mL (95% CI, 15.74–19.16), while those from other regions typically had levels ranging from 12.3 to 14 ng/mL. This regional disparity could be attributed to the warmer weather conditions in South India than in other regions (9).

Harinarayan et al. examined the dietary habits and their relationship with serum calcium and vitamin D [25-hydroxy cholecalciferol 25 (OH) D] levels in residents of Tirupati and surrounding urban and rural villages. Their findings revealed that a considerable segment of the population suffers from low vitamin D levels. About two-thirds (69%) of the studied group showed low levels of vitamin D. Within this group, 15% were diagnosed with vitamin D deficiency (below 20 ng/mL), while 54% had vitamin D insufficiency (ranging from 20 to 29 ng/mL). Notably, all individuals identified with severe deficiency of 25(OH)D (below 5 ng/mL) came from the urban segment of the study population. Significantly higher vitamin D levels were observed in the rural population compared to their urban counterparts. This difference can be partly attributed to the rural population’s greater exposure to sunlight, primarily due to their agricultural occupations (20).

Gandhe et al. studied the levels of circulating 25-hydroxyvitamin D in healthy adolescents and it’s link to body mass index (BMI) in Puducherry. They found that overweight adolescents (BMI 25–29.9) and obese adolescents (BMI ≥ 30) had markedly reduced 25(OH)D levels compared to those with a BMI under 25 (non-obese). Additionally, a strong negative correlation was observed between BMI and serum 25(OH)D levels; as BMI increased, 25(OH)D levels tended to decline (21). It is noteworthy to mention that overweight was common among both patients and controls in our study also. Dietary intake analysis indicated high fat and protein consumption. Overweight among the patients could be due to their limited mobility due to disease related disability. At the same time, in healthy controls, it could be due to sedentary office work and lack of physical activities.

Studies examining vitamin D status in patients with lymphedema other than due to filariasis were also very limited. Ozcan et al. conducted a study comparing vitamin D levels in patients with breast cancer-related lymphedema to those in a healthy control group. The results revealed that 60% of lymphedema patients exhibited vitamin D insufficiency, while 25% had a deficiency, and only 15% maintained adequate vitamin D levels (22). This research is particularly noteworthy, and the results are on the similar lines as observed in the current study on patients with filarial lymphedema. It remains uncertain whether lymphedema leads to vitamin D deficiency.

A review by Tomoka et al. on the effects of vitamin D on immune system and inflammatory diseases found that low serum levels of 25-hydroxyvitamin D (25D) are positively correlated with increased morbidity in upper respiratory tract infections, including flu. The higher infection rate during winter may be partly due to reduced sunlight exposure, which limits the body’s ability to produce the active form of vitamin D. Notably, a 10 nmol/L increase in serum 1,25-dihydroxyvitamin D (1,25D) is associated with a 7% reduction in infection risk (23). Hayashi et al. found that mice fed a high-dose 25-hydroxyvitamin D (25D) diet and then infected with the influenza virus showed a reduction in inflammatory cytokines, specifically IL-5 and IFN-*γ* (24). It is important to point out here that patients with filarial lymphedema suffer from frequent adenolymphangitis attacks and maintaining sufficient vitamin D status might protect them from such episodes.

Some investigators argue for the necessity of developing region-specific reference ranges to accurately evaluate vitamin D status in Indian populations (25). In a study on 270 healthy Indian adults aged between 20 and 50 years revealed that 97.8% had serum 25-hydroxyvitamin D (25HCC) levels falling below the globally recognized threshold of 30 ng/mL (25). Authors report that this benchmark may not be suitable for the Indian demographic. By examining correlations with biochemical markers such as parathyroid hormone (PTH), alkaline phosphatase, and serum phosphate, the researchers recommended lower, population-specific thresholds—designating vitamin D insufficiency at levels under 13.5 ng/mL and deficiency at levels below 7 ng/mL. However, this needs to be validated by large scale population studies in India.

A recent study reports the benefits of calcitriol treatment in experimental lymphedema in rats (26). In this study by Aksöyler et al. when calcitriol was given before and after surgery, it significantly reduced swelling, limited fibrosis, and enhanced healing by promoting anti-inflammatory M2 macrophages and new lymphatic vessel growth. Post-surgery-only treatment showed some benefit, while untreated rats had persistent lymphedema and inflammation. Findings suggest that early calcitriol use may help prevent lymphedema by supporting lymphatic repair and reducing tissue damage (26). This result in animal studies when examined together with the findings of vitamin D deficiency in our study, calls for further research examining the usefulness of vitamin D therapy in human lymphedema cases.

### Limitations of the study

This study has certain limitations, such as a small number of participants and convenient sampling, and the selection bias resulting from the control group comprising of staff from the study site who differ significantly in socioeconomic status and lifestyle from the patient group. Lack of proper sex- and age-matching also adds to the bias. Another limitation of this study is the use of a single 24-h dietary recall to assess nutrient intake. The findings related to micronutrient adequacy should be interpreted with caution. A more robust assessment method, such as a validated food frequency questionnaire (FFQ) or multiple 24-h recalls on non-consecutive days, would have provided a better representation of habitual dietary intake. Future studies should consider these approaches, along with biochemical indicators, to improve the reliability of dietary data and strengthen conclusions about nutrient intake and nutritional status.

## Conclusion

This study reports notable vitamin D deficiency among patients with filarial lymphedema and healthy individuals in South India. This has implications for the MMDP of filariasis elimination program as vitamin D supplementation may be considered in the management protocol for lymphedema. There is a need for similar studies in different eco-epidemiological settings for a better understanding of the extent of the problem and to find the underlying reasons for the deficiency.

In another angle, the high prevalence of deficiency noted among healthy volunteers also calls for action. The need for a balanced diet and adequate sun exposure cannot be overstated. It is also important to create awareness about vitamin D deficiency among health care workers and public alike. Yet another necessity is the studies to define the reference range for defining the deficiency in Indian population as western cut off may not be directly applicable here.

## Data Availability

The original contributions presented in the study are included in the article/supplementary material, further inquiries can be directed to the corresponding author.
